# Fluorimetric detection of reserpine in mouse serum through online post-column electrochemical derivatization

**DOI:** 10.1098/rsos.171948

**Published:** 2018-08-15

**Authors:** Ning Chen, Weixia Li, Shuchao Wu, Yan Zhu

**Affiliations:** 1 Department of Chemistry, Zhejiang University, Xixi Campus, Hangzhou 310028, People's Republic of China; 2 Zhejiang Institute of Geology and Mineral Resources, Hangzhou 310007, People's Republic of China

**Keywords:** reserpine, electrochemical derivatization, serum, online

## Abstract

A novel method combining high-performance liquid chromatography with online post-column electrochemical derivatization and fluorescence detection was established for the detection of reserpine in mouse serum. Reserpine separation was conducted using a C18 column with 5 mM H_3_PO_4_ and acetonitrile (55/45, v/v) as eluent. Reserpine was then electro-oxidized into a strongly fluorescent compound using an electrolytic cell device. Detection parameters, such as potential and fluorescence wavelength, were optimized. The linearity of the proposed method ranged from 0.01 to 5.0 mg l^−1^ with a correlation coefficient of 0.9997. The limit of qualification (*S*/*N* = 10) and limit of detection (*S*/*N* = 3) were 9.7 and 2.9 µg l^−1^, respectively. Resperine recoveries from spiked blank and drug-treated mouse serum samples ranged from 92.0 to 115%.

## 1. Introduction

Reserpine is a naturally occurring indole alkaloid. It was originally isolated from the roots of *Rauvolfia serpentina*. Reserpine was previously used as a primary antihypertensive and antipsychotic agent for the treatment of mild-to-moderate hypertension or vasospastic attacks in Raynaud's phenomenon [1]. However, its use as a primary antihypertensive and antipsychotic agent has been discontinued, given that it can deplete catecholamine in the rodent brain and body [2]. Reserpine overuse can lead to melancholia, as well as result in an array of central nervous system and gastric side effects. Nevertheless, reserpine is used as a second-line adjuvant in combination with other drugs in the treatment of severe forms of hypertension and refractory psychosis. Low doses of reserpine have clinically significant therapeutic effects. The typical initial reserpine dose for humans is 0.5 mg daily for one or two weeks, and the maintenance dose for adults is 0.05–0.25 mg once daily. In rats, the oral administration of 2 g/kg causes sedation and muscle tremors, and intravenous injection with 10 mg kg^−1^ causes death. Overuse may lead to genotoxicity, carcinogenicity and reproductive toxicity (e.g. 1–2 mg kg^−1^ for rats, administered intramuscularly) [3]. Therefore, the detection of reserpine in biological fluids is of urgent importance and interest in clinical analysis.

To date, several works have focused on the analysis of reserpine in tablets [4,5], plants [6–8], plasma [9] and urine [10]. Various methods, such as adsorptive stripping voltammetry [11,12], high-performance thin layer chromatography [13], spectrofluorimetry [14–16], chemiluminescence [17] and capillary electrophoresis [18,19], have been used for reserpine detection. These methods, however, are unsuitable for the online separation and direct detection of reserpine. Thus, high-performance liquid chromatography (HPLC) equipped with UV detector [10,20], photochemical fluorimetry [21] and mass spectroscopy [22,23] have been used for direct reserpine detection. However, the accuracy of UV detection is easily influenced by solvent effect, interferents and matrix complexity; the effects of these factors are particularly robust in biological samples. Thus, a convenient and sensitive reserpine detection system must be established.

Among the above detectors, fluorescence detection exhibits good sensitivity and selectivity. However, reserpine has weak fluorescent properties. Thus, reserpine must undergo pretreatment or post-column derivatization for conversion into a strongly fluorescent compound. These procedures are necessary for reserpine detection with fluorescence detection. Reserpine is converted to 3,4-dehydroreserpine, which exhibits intense green–yellow fluorescence that can be directly detected by a fluorescence detector. Many oxidants, such as nitrous acid [12], cerium (IV) sulfate [16], vanadium pentoxide [24], periodate ion [25] and 2-iodoxybenzoate [26], have been used for reserpine derivatization. Although existing derivatization techniques have their own merits, they are time-consuming and lack sensitivity. Furthermore, samples cannot be simultaneously separated and detected through existing reserpine detection methods. To the best of our knowledge, the fluorimetric detection of reserpine using post-column electrochemical derivatization has not been reported. In addition, the continuous and closed flowing status of online electro-oxidized derivatization avoids baseline noise and instability when the eluent and derivatization agent are mixed together.

In this work, we adapted an HPLC system combined with post-column electrochemical derivatization and fluorescence detector (HPLC/EC/FLD) for the sensitive detection of reserpine. Moreover, we identified the electro-oxidation behaviour of reserpine and optimized the detection parameters of our proposed HPLC/EC/FLD system. When applied to detect reserpine in biological fluids, the proposed system showed remarkable separation, sensitivity and selectivity.

## 2. Experimental

### 2.1. Chemicals and reagents

Powdered reserpine (greater than or equal to 99% purity), dopamine hydrochloride (greater than or equal to 98% purity) and L-dpoa (greater than or equal to 99% purity) were purchased from Aladdin Bio-Chem Technology Co., Ltd (Shanghai, China). HPLC-grade acetonitrile (ACN), phosphoric acid (H_3_PO_4_) and other chemicals were obtained from Huipu Chemical Reagent Co., Ltd (Hangzhou, China). All aqueous solutions were prepared using deionized water purified with Milli-Q system (Millipore, Bedford, MA, USA) with a specific resistivity of 18.2 MΩ cm.

### 2.2. Solution

Reserpine stock solution was prepared with a concentration of 200 mg l^−1^ by dissolving 10 mg of reserpine in 50 ml ACN in a brown volumetric flask. A series of working solutions containing the concentration of 0.01, 0.02, 0.05, 0.1, 0.5, 1.0 and 5.0 mg l^−1^ was diluted with the HPLC mobile phase (supporting electrolyte). The stock solution was stored in a refrigerator at 4°C, and working standard solutions were freshly prepared every day.

### 2.3. Equipment

#### 2.3.1. Apparatus and chromatographic conditions

**Figure 1.**
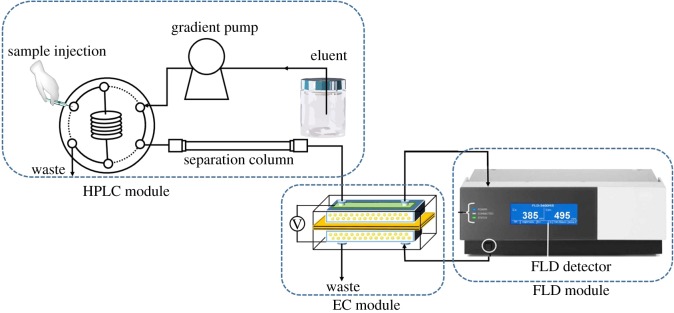
Schematic of HPLC/EC/FLD system.

The schematic of the complete HPLC/EC/FLD system is shown in figure 1. HPLC analysis was performed with UltiMate-3000 (Thermo Fisher Scientific, USA) equipped with a quaternary gradient pump, a six-port valve fitted with a 25 µl injection loop and a thermal column compartment. Fluorescence signals were recorded with an FLD-3100 fluorescence detector (Thermo Fisher Scientific). Data collection and analysis were performed with Chromeleon 7.2.SR5 workstation (Thermo Fisher Scientific). Centrifugation and precipitation for sample pretreatment were performed with a Lab LD5-10B centrifuge (Beijing, China).

The mobile phase consisted of a mixture of ACN and 5 mM H_3_PO_4_ aqueous solution (45/55, v/v) pumped at a flow rate of 1.0 ml min^−1^. The injection volume was 25 µl. An Acclaim 120 C18 column (250 × 4.0 mm i.d.) was used as the analytical column.

#### 2.3.2. Fabrication of the electrolytic cell device

**Figure 2.**
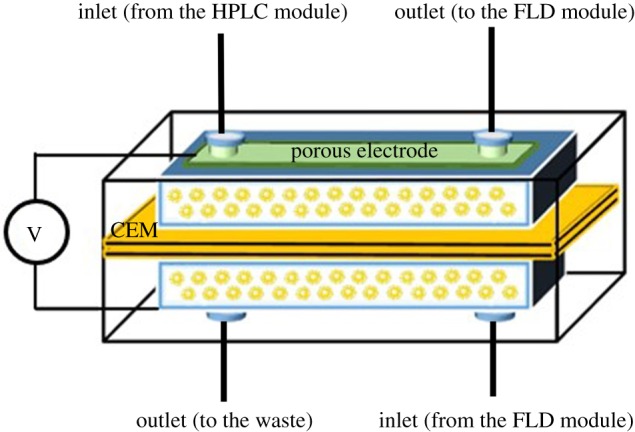
Structure of the electrolytic cell device. CEM, cation-exchange membrane.

The structure of the electrolytic cell device (EC) is shown in figure 2. The EC consisted of two rectangular polytetrafluoroethylene (PTFE) blocks (92** ** × 40** ** × 12** **mm), two chambers (56** ** × 14** ** × 1** **mm) machined with size-matching porous Ti electrodes (56** ** × 14** ** × 0.5** **mm, Qingdao Shenghan Chromatography Tech Equipment Co., Ltd, Qingdao, China) and a cation-exchange membrane (92** ** × 40** ** × 0.5** **mm, Shanghai Chem. Co., Shanghai, China). Screws were used to immobilize PTFE blocks symmetrically and tightly (not shown in figure 2). The flow route of the eluent (supporting electrolyte) via four inlets was assured. The power supply (with the resolution of 0.1** **V) was provided by power supply rectifiers (Xianke Scientific Instrument Co., Taishan, China).

### 2.4. Preparation of mouse serum

Mouse serum samples were provided by the Zhejiang Provincial Tongde Hospital (Hangzhou, China). Each participant weighted around 25** **g was decapitated and the blood was collected and centrifuged for reserve after the intraperitoneal injection of 0.5 ml reserpine.

Mouse serum samples were prepared through protein precipitation. First, 0.8 ml acetonitrile and 0.2 ml mouse serum were mixed. The mixture was then centrifuged at 3000** **r.p.m and 4°C for 15** **min. The supernatant was filtered through a 12** **mm membrane syringe filter (Xiboshi, pore size 0.45 µm, Tianjin Fuji Tech Co., Tianjin, China) prior to injection into the system.

### 2.5. Method validation and recovery

The absolute recovery of the drug from serum was quantified. Briefly, 1 ml samples of blank serum were spiked with reserpine at 50, 100 and 500 µg l^−1^ concentrations. The peak areas of the samples after chromatographic and electrochemical derivatization were compared with those of working standard aqueous solutions of reserpine at the same concentrations.

## 3. Result and discussion

### 3.1. Electrochemical derivatization of reserpine

The schematic of the complete HPLC/EC/FLD system procedure is shown in figure 1. In this system, reserpine is electro-oxidized after liquid chromatographic separation and is then directly detected by a fluorescence detector.

Reserpine dehydrogenation is already well known among analytical chemists [27–29]. Here, we selected reserpine as the target analyte, given its weak natural fluorescent property. Reserpine contains available nitrogen with an unshared pair of electrons. A two electron oxidation occurs attributed to the formation of an N-oxide [30]. In the proposed method, reserpine is converted to a π-electron-rich compound when electro-oxidized through the irreversible electron-transfer reaction [11]. The long conjugate structure of this compound and free radicals strengthen fluorescence signals. Thus, a product with intense green–yellow fluorescence is formed through the oxidation of reserpine under optimized conditions (e.g. solution medium and potential). This strongly fluorescent product is easily detected through fluorimetry.

**Figure 3.**
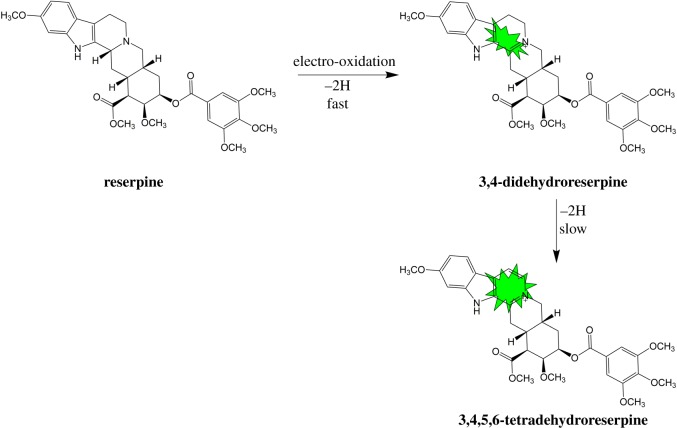
Electro-oxidation mechanism of reserpine.

Sánchez & Sánchez-Aibar [16] reported that reserpine is quickly converted to 3,4-dehydroreserpine (excitation and emission maxima of 390 and 490** **nm, respectively), whereas 3,4,5,6-tetradehydroreserpine (excitation and emission maxima of 330 and 435** **nm, respectively), a second-step oxidation product, is formed slowly. These oxidation products have been examined through electrospray ionization mass spectrometry [28–31]. Figure 3 shows the electro-oxidation mechanism of reserpine and the luminescent groups (the region marked in green).

**Figure 4.**
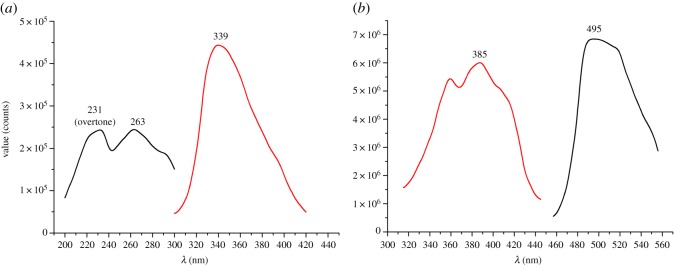
(*a*) Excitation spectra and emission spectra of reserpine. Experimental condition: *E* = 0 V; (*b*) excitation spectra and emission spectra of electro-oxidized product. Experimental condition: *E* = 0.9 V.

The fluorescence properties of the product are of interest for analytical purposes. We attempted to identify the excitation and emission wavelength of the products through full rapid scanning under an unsegmented continuous-flow status (without connecting the column, as shown in electronic supplementary material, figure S1). Optimization was performed with the EC turned on and off successively. We thus obtained the chromatograms (figure 4) of these compounds. The maximal excitation and emission wavelengths of reserpine were 231** **nm (overtone) and 263/339** **nm, respectively, and the emission wavelength of the electro-oxidized product was 385/495** **nm. Based on excitation and emission maxima which were consistent with previous works, our results suggested that the product is 3,4-dehydroreserpine [14–16]. The comparison between the chromatograms of reserpine and its electro-oxidation product is shown in electronic supplementary material, figure S2. In addition, post-column derivatization occurs under continuous-flow states, the fluorescence of 3,4-dehydroreserpine can be easily detected.

### 3.2. Optimization of the chromatographic separation system

Recent works have detected reserpine through the HPLC method with an aqueous solution and organic modifier (methanol or acetonitrile) as the mobile phase. Our proposed HPLC/EC/FLD system, which uses the mobile phase described in §2.3.1, could elute reserpine within 12** **min.

The eluent served as the supporting electrolyte in the post-column electrochemical derivatization. Thus, ACN was maintained at a relatively low concentration to ensure the sufficient conductivity of the supporting electrolyte. Calatayud & Benito [32] pointed out that acetic acid is the most favourable medium for the photochemical reaction of reserpine. Stankovic *et al*. [33] found that an acidic medium favours the electrochemical detection of reserpine. Given that the H_3_PO_4_ environment contributes to fluorescence signals (electronic supplementary material, figure S2), we selected H_3_PO_4_ as the reaction medium for the online electrochemical reaction of reserpine. A mixture of 45% (v/v) ACN and 5** **mM H_3_PO_4_ was used as the elute/supporting electrolyte, which shortened the retention time and intensified the fluorescence signal of reserpine. These results are in accordance with the principle stating that increasing the concentration of the supporting electrolyte promotes oxidation efficiency [34].

Furthermore, the addition of H_3_PO_4_ improved peak shape. As H_3_PO_4_ concentration reached 5** **mM, the peak curve and the retention time of reserpine remained stable. Considering the above phenomena, we selected the 55 : 45 (v/v, pH = 2.6) mixture of 5** **mM H_3_PO_4_ and ACN as the optimal eluent. The results of a tablet test indicated that reserpine could be separated from other interferents using this eluent.

### 3.3. Optimization of high-performance liquid chromatography system combined with post-column electrochemical derivatization and fluorescence detector

We optimized several parameters of the HPLC/EC/FLD procedure. All parameters were independently varied and were considered optimized when the peak area (or height) of reserpine reached the maxima.

#### 3.3.1. pH response of reserpine oxidation

**Figure 5.**
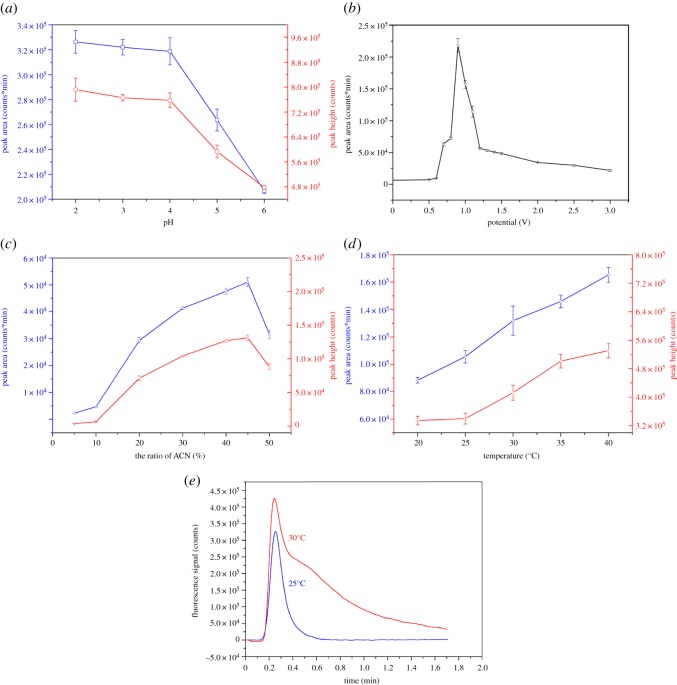
Optimization: (*a*) dependence of the peak area and peak height on pH; (*b*) effect of potential on the fluorescence signal of reserpine (5 mg l^−1^); (*c*) dependence of the peak area and peak height on ACN ratio; (*d*) dependence of the peak area and peak height on temperature; (*e*) chromatograms of electro-oxidized reserpine with different temperature.

pH is another factor that affects reserpine oxidation. We investigated the effect of this parameter by using the manifold shown in electronic supplementary material, figure S1. ACN was maintained at the same ratio (45%). The influence of pH within the range of 2–6 on the peak area (height) was obtained and is shown in figure 5*a*. Peak area (height) decreased insignificantly within pH 2–4. A drastic decrease in the signal was found, when pH exceeded 4. The dissociation constant (pKa) of reserpine is 6.6 (at 25°C) [35]. Calculations revealed that only at pH less than 4.6 can reserpine dissociate completely. This result was consistent with the pH dependence found in the present work, where the slopes changed abruptly between pH 4 and 5. Incomplete dissociation of reserpine resulted in drastically decreased fluorescence signal at pH greater than 4.6 (figure 5*a*). Stankovic *et al*. [33] reported that protons participated in the electro-oxidation of reserpine, in agreement with the pH response that insignificantly decreased within pH 2–4.

The pH of 5** **mM H_3_PO_4_ is 2.6. Reserpine dissociated completely, and electrochemical oxidation remained almost unchanged with pH 2–4. Therefore, 5** **mM H_3_PO_4_ with 45% (v/v) of ACN was selected as the eluent, considering the service life of C18 column and the easy operation.

#### 3.3.2. Potential of the electrolytic cell device for electrochemical derivatization

The efficient electro-oxidation of reserpine is a critical factor of the final detection limit of the proposed detection system. Therefore, the potential of the EC should be optimized.

We investigated the relationship between the potential of the EC and the fluorescence signal of reserpine. We used the mobile phase discussed in §2.3.1 as the supporting electrolyte. We varied the potential of the EC from 0 to 3.0** **V. As shown in figure 5*b*, the fluorescence signal dramatically intensified and reached its maximum at the potential of 0.9** **V. This behaviour may have resulted from the generation of 3,4-dehydroreserpine, which is strongly fluorescent. However, the fluorescent signal remarkably decreased when the potential exceeded 0.9** **V. This phenomenon might be attributed to some side effects and the products might yield weaker fluorescence intensity compared with that of 0.9** **V.

Therefore, we selected 0.9** **V as the optimal potential.

#### 3.3.3. Acetonitrile ratio in the mobile phase

The results of our preliminary tests, which were conducted under flowing status, confirmed that the online electrochemical reaction is related to the addition of ACN. To date, no work has found that ACN contributes to the electrochemical reaction of reserpine. We further investigated the influence of the ratio of ACN (figure 5*c*) on the electrochemical reaction of reserpine. Increasing the ratio of ACN intensified the fluorescence signal of reserpine after the background/solvent signal was subtracted. The luminescent groups of reserpine are hydrophobic, and the added proportion of ACN in the eluent may have promoted the exposure of luminescent groups. This effect, in turn, may have promoted the electrochemical reaction. However, the fluorescence signal decreased when ACN ratio was increased to 50%. As we mentioned previously, the mobile phase serves as the supporting electrolyte. Thus, potential should be increased when ACN ratio is higher than 45%.

We selected 45% (v/v) ACN for optimal fluorescence signalling and separation efficiency.

#### 3.3.4. Temperature

Moreover, we investigated temperature as a detection parameter of the proposed HPLC/EC/FLD method. Increased environmental temperature decreased solution viscosity but increased molecular motion frequency, thus intensifying the fluorescence signal (figure 5*d*). However, high temperatures aggravated molecular diffusion, which caused strong peak tailing (e.g. as shown in figure 5*e*).

Given these considerations, we selected 25°C as the optimal temperature.

To obtain the best results, 5** **mM H_3_PO_4_ (pH = 2.6) with 45% (v/v) of ACN was selected as the eluent at 25°C and for a value of the applied potential of 0.9** **V.

### 3.4. Chromatographic performance and validation of the procedure

#### 3.4.1. Linearity

HPLC/EC/FLD was used to detect reserpine in an external standard solution. The peak area obtained under the optimized detection conditions was measured at least thrice. The linear equation of reserpine is *y* = 14670*x* + 761 with the range of 0.01–5.0** **mg l^−1^ with a correlation coefficient (*r*^2^) of 0.9997. The limit of quantification (based on signal-to-noise ratio of 10, *S*/*N* = 10, electronic supplementary material, figure S3) was 9.7 µg l^−1^, and the limit of detection (LOD) (*S*/*N* = 3) was 2.9 µg l^−1^. These results indicated that our proposed method exhibits good sensitivity and repeatability.

#### 3.4.2. Accuracy and precision

We investigated the accuracy of the proposed method using samples spiked with precisely weighed amounts of reserpine. We interpolated spiked recoveries from calibration curves. We obtained recovery values of 92.0 to 115%, indicating that our proposed method has good accuracy. Furthermore, the ultraviolet spectra of the analyte confirmed the analyte was reserpine, instead of its metabolites (electronic supplementary material, figure S4).

We examined the precision of the proposed method by using three duplicate samples (table 1). The RSDs of peak areas were less than 2% for the analysis of a standard solution containing 2** **mg l^−1^ reserpine. For matrix-matched analysis, the RSDs were less than 6.4% in table 1. These results confirmed the good precision and stability of our proposed method.

**Table 1.** Recoveries of spiked reserpine and assay precision for reserpine detection in mouse serum.

sample	found (mg l^−1^)	precision (%R.S.D., *n* = 3)	spiked (mg l^−1^)	detected (mg l^−1^)	recovery (%)	precision (%R.S.D., *n* = 3)
sample 1	1.02	3.0%	0.100	1.16	105	1.4
			intra-day			
sample 2			0.050	0.048	96.2	6.4
	/	/	0.100	0.111	111	3.9
			0.500	0.575	115	1.4
			inter-day			
			0.050	0.046	92.0	3.5
			0.100	0.095	95.1	5.3
			0.500	0.571	114	5.5

Sample 1: drug-treated mouse serum group.

Sample 2: blank control serum group.

#### 3.4.3. Comparison with previously reported methods

We compared the performance of the proposed method in reserpine detection with those of previously reported works. We summarized the comparisons in table 2. Our proposed method has good sensitivity. The proposed method shows that it is appropriate for detection in serum considering the linear range of the method and the typical levels of reserpine in serum. By contrast, other methods showed narrow linear ranges or high detection limits. These properties limit the application of other methods in reserpine detection in biological fluids. We attributed the good sensitivity of our method to effectual electro-oxidized approach and proper choice of detector. Compared with the electrochemical method [12] which shows similar linear range and detection limits; however, the proposed method shows advantage in terms of selectivity. In addition, the detection limit of our proposed method is lower than those of other methods, except for that of tandem mass spectrometry. However, fluorescence spectrometry has superior reproducibility, easier operation, lower economic cost and better selectivity than tandem mass spectrometry. Thus, our present approach is sensitive and selective compared with other approaches reported. A further applicability of our present approach for analysis in serum is investigated.

**Table 2.** Comparison of the proposed method with other reported methods. SECNT/CPE-modified PANI, single-walled carbon nanotube paste electrode modified with polyaniline; MWNT, modified GCE, multi-walled carbon nanotube-modified glassy carbon electrode; FI, flow injection; LC, liquid chromatography.

methods	sample	treatment	detector	linearity range (mg l^−1^)	LOD (μg l^−1^)	ref.
HPLC	mouse serum	post-column electrochemical derivatization	FLD	0.01–5.0	2.9	present work
HPLC	herbs	drying, dissolution and extraction	UV	1.56–200	390	[20]
capillary electrophoresis	*Rauvolfia yunnanensis*	extraction, centrifugation and concentration	DAD	10–100	500	[19]
capillary electrophoresis	urine	filtration	ECL	0.608–60.8	42.60	[18]
chemiluminescence	tablet	oxidation with potassium permanganate	chemiluminometer	0.100–3.00	48	[17]
adsorptive stripping voltammetry with SWCNT/CPE-modified PANI	plant sample	drying, acid dissolution and concentration	ECD	0.085–0.87	0.407	[11]
adsorptive stripping voltammetry with MWNT-modified GCE	tablet and injection	100-fold dilution	ECD	0.012–6.08	4.5	[12]
FI photochemical spectrofluorimetry	tablet	acetone sensitization	spetrofluorimeter	0.01–0.75	0.45	[15]
LC	equine plasma	liquid–liquid extraction solid-phase extraction	MS/MS	0.010–5.0 μg l^−1^; 0. 100–5.0 μg l^−1^	0.01	[23]
HPLC	human plasma	liquid–liquid extraction	MS/MS	0.04–30.00 μg l^−1^	/	[22]

Porous Ti electrode was used as the working electrode for efficient electrochemical derivatization. Compared with some other electrodes such as gold and Pt electrodes, the porous Ti plate is cheap, commercially available and electrochemically stable and is thus an excellent electrode for flowing electrochemical reactions. Porous Ti electrode reportedly has an effective surface area four times larger than that of planar Ti electrode [36,37] and was selected as the working electrode in the present study. The electro-oxidation of reserpine can occur in the interior and the surface of the porous electrode. Strong oxidation capacity can be obtained, thereby greatly improving the detection sensitivity.

#### 3.4.4. Analysis of reserpine in mouse serum samples

**Figure 6.**
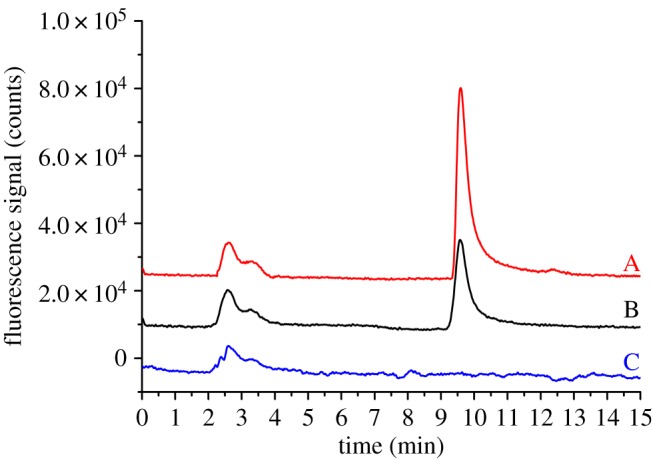
Chromatograms of reserpine in mouse serum as obtained through the HPLC/EC/FLD system. (A) Blank mouse serum sample spiked with 2 mg l^−1^ reserpine, (B) drug-treated mouse serum sample and (C) blank control mouse serum sample. Column: Acclaim 120 C18 column. Eluent: 5 mM H_3_PO_4_ and ACN (55/45, v/v).

We successfully applied the optimized HPLC/EC/FLD method in the detection of reserpine in mouse serum samples. The detection of reserpine in real biological samples is complicated by the complexity and diversity of biological samples. The standard addition curve was in the range of 0.05–5.0** **mg l^−1^ reserpine, with *r*^2^ of 0.9997 for serum. The slopes of the external standard curve and standard addition curve were similar (14 670 and 12 977, respectively, as shown in electronic supplementary material, figure S5). Spiked recoveries ranged from 92.0 to 115%. Statistical parameters are presented in table 1. For comparison, blank and drug-treated mouse serum was tested. The chromatograms of mouse serum samples are shown in figure 6. Other related alkaloids with similar structure (e.g. rauwolscine, sarpagine, yohimbine) were completely non-fluorescent at the maximal wavelength of 3,4-dehydroreserpine [38]. No interferences (such as dopamine, L-dopa, 1** **ppm) were observed (electronic supplementary material, figure S6).

## 4. Conclusion

We proposed a sensitive and selective approach that combines chromatographic separation and post-column derivatization for reserpine detection. We optimized the eluent, potential and wavelength parameters of our proposed method to induce the emission of strong fluorescent signals from reserpine. Reserpine can be directly detected after separation with the C18 column and electro-oxidation. The proposed method has the merits of good separation and low detection limit. We successfully applied our proposed method in the detection of reserpine in mouse serum. Results indicated that our method has potential applications for reserpine detection in other biological fluids.

## Supplementary Material

Supporting Information

## Data accessibility

This article does not contain any additional data.
