# Supplementary material for: Fluorimetric detection of reserpine in mouse serum through online post-column electrochemical derivatization
Source: R Soc Open Sci. 2018 Aug 15;5(8):171948. doi: 10.1098/rsos.171948 (PMC6124075; doi:10.1098/rsos.171948)
Supplement: Supporting Information [file rsos171948supp1.docx]

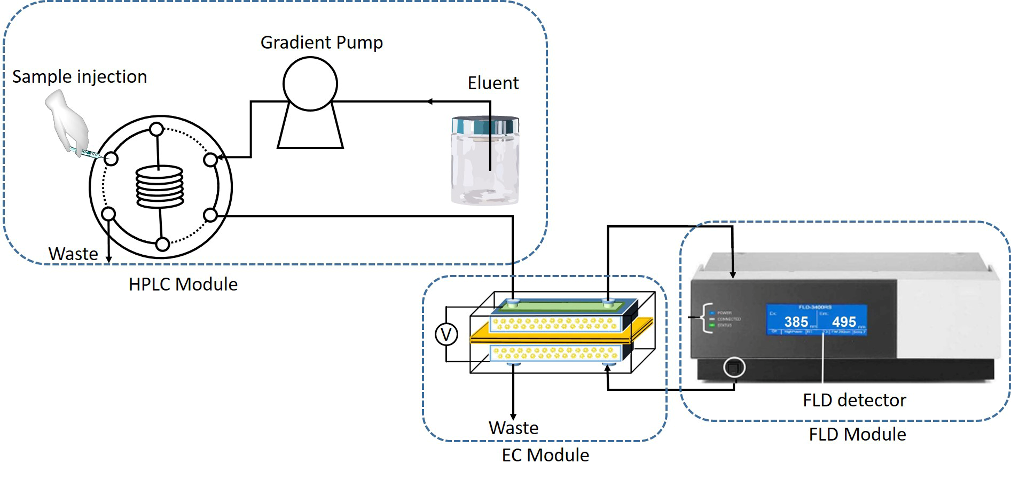


**Figure S1** Schematic of an unsegmented continuous-slow status without connecting the column

^

^

**Figure S2** Effect of potential on the the fluorescence signal of reserpine. Experimental condition: λex/λem=385/495 nm





**Figure.S3** Chromatograms of the limit of quantitation.





**Figure.S4** Ultraviolet Spectra of the analyte.





**Figure.S5** Linearity of (A) external standard solution (B) standard spiked serum.





**Figure. S6** Chromatograms of (A) reserpine, (B) reserpine, dopamine and L-dopa, (C) dopamine, (D) L-dopa.
